# Extremely preterm children exhibit increased interhemispheric connectivity for language: findings from fMRI‐constrained MEG analysis

**DOI:** 10.1111/desc.12669

**Published:** 2018-04-16

**Authors:** Maria E Barnes‐Davis, Stephanie L Merhar, Scott K Holland, Darren S Kadis

**Affiliations:** ^1^ Perinatal Institute Cincinnati Children's Hospital Medical Center Cincinnati Ohio USA; ^2^ Department of Pediatrics University of Cincinnati Cincinnati Ohio USA; ^3^ Pediatric Neuroimaging Research Consortium Cincinnati Children's Hospital Medical Center Cincinnati Ohio USA; ^4^ Department of Radiology and Medical Imaging Cincinnati Children's Hospital Medical Center Cincinnati Ohio USA; ^5^ Division of Neurology Cincinnati Children's Hospital Medical Center Cincinnati Ohio USA

## Abstract

Children born extremely preterm are at significant risk for cognitive impairment, including language deficits. The relationship between preterm birth and neurological changes that underlie cognitive deficits is poorly understood. We use a stories‐listening task in fMRI and MEG to characterize language network *representation* and *connectivity* in children born extremely preterm (*n* = 15, <28 weeks gestation, ages 4–6 years), and in a group of typically developing control participants (*n* = 15, term birth, 4–6 years). Participants completed a brief neuropsychological assessment. Conventional fMRI analyses revealed no significant differences in language network representation across groups (*p* > .05, corrected). The whole‐group fMRI activation map was parcellated to define the language network as a set of discrete nodes, and the timecourse of neuronal activity at each position was estimated using linearly constrained minimum variance beamformer in MEG. Virtual timecourses were subjected to connectivity and network‐based analyses. We observed significantly increased beta‐band functional connectivity in extremely preterm compared to controls (*p* < .05). Specifically, we observed an increase in connectivity between left and right perisylvian cortex. Subsequent effective connectivity analyses revealed that hyperconnectivity in preterms was due to significantly increased information flux originating from the right hemisphere (*p* < 0.05). The total strength and density of the language network were not related to language or nonverbal performance, suggesting that the observed hyperconnectivity is a “pure” effect of prematurity. Although our extremely preterm children exhibited typical language network architecture, we observed *significantly altered network dynamics*, indicating reliance on an alternative neural strategy for the language task.

**RESEARCH HIGHLIGHTS**

- Conventional task‐based fMRI analyses suggest that the *spatial distribution of the receptive language network is unchanged* in children born <28 weeks gestation.
- MEG source‐space connectivity analyses reveal that the *dynamics of the language network are significantly altered* in children born extremely preterm.
- Children born extremely preterm exhibit significantly increased connectivity between left and right perisylvian cortex, compared to term controls.
- Effective connectivity analyses reveal that hyperconnectivity in preterms is due to increased information flux originating from the right hemisphere.

## 1 INTRODUCTION

Neurocognitive deficits in children born preterm are well established. Language and cognition are inextricably linked, and extremely preterm (EPT) infants are vulnerable to disruption of simple and complex language functions (Barre, Morgan, Doyle, & Anderson, 2011; Crosbie, Holm, Wandschneider, & Hemsley, 2011; van Noort‐van der Spek, Franken, & Weisglas‐Kuperus, 2012; Vohr, 2014). Single modalities, such as structural imaging at term and early language testing, leave a large proportion of the variance in later cognitive functioning unexplained (Luttikhuizen dos Santos, de Kieviet, Konigs, van Elburg, & Oosterlaan, 2013; Woods, Rieger, Wocadlo, & Gordon, 2014). Thus, it is important to study NICU graduates beyond 3 years of age to observe maturing functional language networks and to incorporate multiple testing modalities.

The majority of neuroimaging studies in preterm children are structural in nature, finding smaller overall brain volumes associated with global intelligence, language performance, and executive functioning (Abernethy, Palaniappan, & Cooke, 2002; Cheong et al., 2013; de Kieviet, Zoetebier, van Elburg, Vermeulen, & Oosterlaan, 2012; Isaacs et al., 2004; Kidokoro, Neil, & Inder, 2013; Lowe et al., 2011; Lowe et al., 2013; Omizzolo et al., 2013). Structural imaging provides insights into brain injuries associated with prematurity presumed to underlie neurocognitive dysfunction, but cannot provide information regarding brain activation in response to specific demands. Functional MRI (fMRI) is the preferred approach for language mapping. It is noninvasive, has excellent spatial resolution, and clinically validated protocols for lateralization of receptive and expressive language are readily available.

Studies investigating brain networks supporting language and the impact of prematurity on these networks are few. Those utilizing task‐based fMRI suggest that preterm children and adolescents have atypical representation in language networks, relying on more diffuse language pathways including areas in the dorsal right hemisphere and increased bitemporal connectivity, but association with performance is inconsistent (Gozzo et al., 2009; Kwon et al., 2015; Kwon et al., 2016; Myers et al., 2010; Schafer et al., 2009; Scheinost et al., 2015; Wilke, Hauser, Krageloh‐Mann, & Lidzba, 2014). Prior studies relied on fMRI functional connectivity in older children and adolescents and did not give information regarding directionality of connections. They also included children with overt brain injury or neurological deficits, which calls into question if this is a “pure” effect of altered development in prematurity or due to associated brain injury.

Despite its strengths, fMRI lacks the fine temporal resolution necessary to characterize fast neuronal activity. Everyday language functions rely on the rapid integration of processes supported by an anatomically diffuse network of regions (see for example, Price, 2012). Magnetoencephalography (MEG) offers sub‐millisecond temporal resolution, permitting relatively direct assessment of neuronal activity and access to the fast network dynamics. MEG is also silent, and in our experience, children report less discomfort and feelings of claustrophobia than in MRI. As such, MEG is especially suited to studying the cortical reorganization of language networks in children born preterm.

MEG has been used to study auditory and phonological processing in‐utero and language networks in term children (Guzzetta, Conti, & Mercuri, 2011; Kadis, Dimitrijevic, Toro‐Serey, Smith, & Holland, 2016; Muenssinger et al., 2013; Sheridan et al., 2010) and established spatial concordance with fMRI for language (Pang, Wang, Malone, Kadis, & Donner, 2011; Wang, Holland, & Vannest, 2012). It has not previously been used to study language networks in preterm children. In this study, we use fMRI and MEG to examine language networks in extremely preterm children at 4–6 years of age as compared with term controls, and relate network measures to language performance.

We theorize that the developing brain has plastic potential to support language. The network is impacted by prematurity even in the absence of overt brain injury, particularly when the brain experiences the entire third trimester of gestation (a time of avid synaptogenesis and pruning; Tau & Peterson, 2010) in an extra‐uterine environment. We hypothesize prematurity disrupts normal language network development leading to increased reliance on alternative pathways, including persistent right hemisphere connectivity. We investigate whether such alteration in distribution and connectivity of language networks occurs in children born <28 weeks of gestation, using functional neuroimaging to describe the preterm network supporting language and determine if it represents a compensatory mechanism that improves language outcomes as indexed by standardized neurobehavioral assessments of language.

## 2 METHODS

### 2.1 Participants

This is an observational study with 30 participants recruited from the greater Cincinnati area. Extremely preterm (EPT, *n* = 15) children were recruited through the Neonatal Research Network Low Birth Weight Follow‐Up Study using phone calls and letters. Term controls (TC, *n* = 15) were recruited through Cincinnati Children's Hospital Medical Center research opportunity advertisements.

This cohort of 30 children provides adequate power (0.8) to detect between‐group differences in accordance with our *a priori* calculations and similar studies in pediatric populations (Frye et al., 2009; Frye et al., 2010; Horowitz‐Kraus, Vannest, Gozdas, & Holland, 2014; Kadis et al., 2011; Pang et al., 2011; Peterson et al., 2002; Youssofzadeh, Williamson, & Kadis, 2017). Fifteen 4–6‐year‐old children who were born at term (37–42 weeks gestation) were recruited from the community and comprised the control group. Fifteen 4–6‐year‐old children who had participated in the NICHD Neonatal Research Network Low Birth Weight Follow Up Study were recruited by invitation if they were born at <28 weeks gestation, had no grade 3–4 IVH on neonatal cranial ultrasound, and had Bayley scores within normal range at 2 years. Children with cerebral palsy, seizures, migraines, history of learning or speech disability, or history of speech therapy were excluded from both groups. The study was approved by Cincinnati Children's Hospital IRB and conforms to the US Federal Policy for the Protection of Human Subjects. Consent was obtained from parents and assent from all children.

### 2.2 Behavioral methods

Children underwent limited cognitive assessment, including the Peabody Picture Vocabulary Test (PPVT4) (Dunn, Dunn, & Lenhard, 2015), Expressive Vocabulary Test (EVT2) (Williams, 2007), Wechsler Nonverbal Scale of Ability (WNV) (Wechsler & Naglieri, 2006), and Edinburgh Handedness Inventory (EHI) (Oldfield, 1971). They then participated in MEG scanning followed by structural MRI and fMRI on the same day.

### 2.3 Stimuli

The receptive language paradigm has been extensively used, consisting of five stories developed at our center by a speech language pathologist, targeted for this age range and presented aurally in a female voice (Holland et al., 2007). Children listened to the same story stimuli during MEG and fMRI. In fMRI, stories were presented in a 32 second block. In MEG, the same stories were presented as individual sentences of approximately 2–3 seconds in duration. Between story stimuli presentation, children listened to speech‐shaped noise, matched to the story stimuli for duration, spectral content and amplitude envelope.

### 2.4 MRI acquisitions

All subjects were studied awake without sedation, as our center has vast experience in neuroimaging across the lifespan, including young children, without pharmacologic assistance. We conducted fMRI scanning at 3T (Philips Achieva scanner, TR/TE = 2000/30ms, flip = 75°, 2.8 × 2.8 × 3.0 mm voxels). We obtained 3D T1‐weighted volumes at 1.0 × 1.0 × 1.0 mm. To facilitate MEG‐MRI coregistration (required for accurate forward modeling in source analyses), radiographic markers were placed at nasion and preauricular positions prior to acquiring images.

### 2.5 MEG acquisition

MEG data were acquired on a 275‐channel whole‐head CTF system (MEG International Services Ltd., Coquitlam, BC). We recorded neuromagnetic activity at 1200 Hz. Subjects were tested while supine, listening to stimuli via a calibrated audio system comprising distal transducer, tubing, and ear inserts (Etymotic Research, IL, USA). Head localization coils were placed at nasion and preauricular locations. Total head displacement was <5 mm from beginning to end of acquisition.

### 2.6 fMRI analyses

fMRI data were analyzed using a conventional general linear model (GLM) approach in SPM12 (http://www.fil.ion.ucl.ac.uk/spm/software/spm12/) running in Matlab R2014b (MathWorks Inc., Natick, MA). Preprocessing entailed realignment, coregistration to individual subject's T1‐weighted images, spatial normalization to template space (MNI 152), and smoothing. Interscan movement was quantified during realignment; motion parameters (six per acquisition) were entered into first‐level analyses as nuisance regressors. Contrast maps were generated (stories minus noise) for each subject, and passed on for subsequent group comparisons (second‐level analysis). At the second level, we compared the spatial distribution of language maps for children born preterm and term controls.

**Figure 1:**
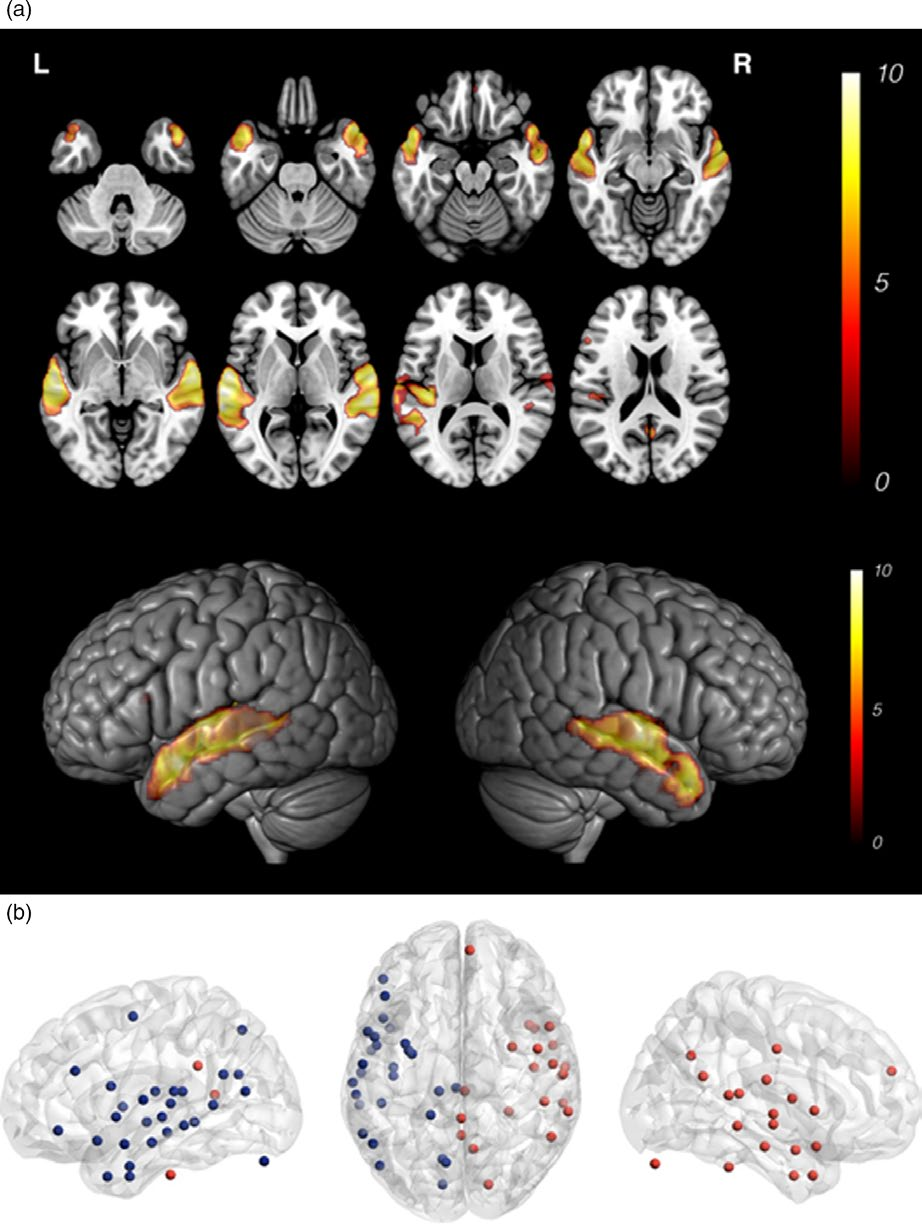
Functional MRI activation maps and network nodes. (A) Eight axial slices showing typical bilateral temporoparietal activation for stories versus noise (*p* < .001, *n* = 30, 15 TC and 15 EPT) and 3D surface rendering showing activation from lateral perspective. (B) The activation map is parcellated to produce the “nodes” for network analysis

To objectively identify the language network in our study cohort, we computed the joint activation map for children born preterm and typically developing controls. The resulting activation map was sectioned using a 200‐unit random parcellation scheme (Craddock, James, Holtzheimer, Hu, & Mayberg, 2012). Centroids of parcels with significant activation on fMRI served as network nodes for subsequent MEG virtual sensor extraction and connectivity analyses (Figure 1B) (Kadis et al., 2016).

### 2.7 MEG preprocessing and virtual sensor extraction

We computed the timecourse for activity at each node location, and subjected these to functional and effective connectivity analyses. MEG data were analyzed using FieldTrip routines (open‐source Matlab toolbox for analyses of electrophysiological data) (Oostenveld, Fries, Maris, & Schoffelen, 2011). Continuous neuromagnetic data were initially bandpass filtered from 0.1 Hz to 100 Hz, and 60 Hz power‐line noise attenuated using a sharp discrete Fourier transform filter. The data were then segmented into 48 epochs of interest (0–2000 ms from onset of sentences). Scanner jump artifacts were automatically detected, and trials containing artifact were rejected from each dataset. We required at least 50% trial retention for inclusion in subsequent analyses. Across participants, datasets included a mean of 46.4 of 48 trials (standard deviation = 4.7). Realistic single‐shell headmodels were constructed from individual 3D T1‐weighted images. A 3D grid was constructed with dipole resolution of 5 mm on a template (MNI151) brain volume. The template and grid were warped to individual anatomical space, prior to inversion. Using a linearly constrained minimum‐variance beamformer (LCMV) with 0.1% regularization, we estimated the time series of activity at each network node (virtual sensor analysis).

### 2.8 MEG functional connectivity analyses

**Figure 2:**
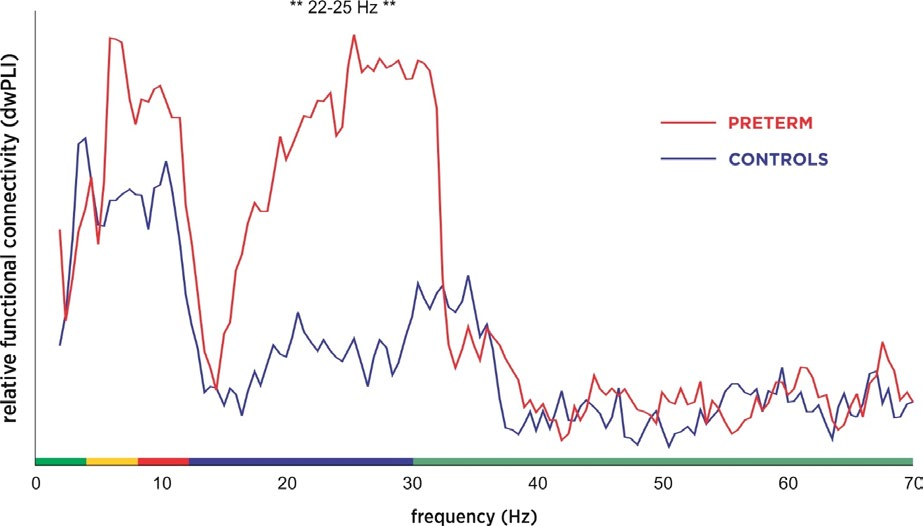
Functional connectivity indexed by debiased weighted phase lag index for each group. Debiased weighted phase lag index (wPLI) for extremely preterm children (EPT,* n* = 15, shown in red) and term controls (TC,* n* = 15, in blue). Areas of significant difference are marked with asterisks

We computed pairwise functional (undirected) connectivity within the network using debiased weighted phase‐lag index (wPLI) in broadband (2–70 Hz), for each group. Weighted phase lag index (PLI) is a measure of phase difference distribution across trials; consistent phase differences are reflected in greater wPLI values, indicating nontrivial functional connectivity between a pair of nodes (Vinck, Oostenveld, van Wingerden, Battaglia, & Pennartz, 2011). We visualized the connectivity spectra for each group, identifying frequencies of interest. Simple between‐group *t* tests revealed a contiguous frequency band of significantly increased functional connectivity (22–25 Hz, high‐beta band, Figure 2) in preterms. We computed mean functional connectivity within the 22–25 Hz frequency band for each subject, and evaluated group differences in network extent using the Network Based Statistics (NBS) (Zalesky, Fornito, & Bullmore, 2010). NBS was employed to evaluate mass univariate testing of connections within the network, across a range of (arbitrary) initial thresholds. Supra‐threshold connections are clustered in topological space and subjected to permutation testing. To determine whether the language network connectivity was related to performance, we computed total network strength (sum of absolute debiased wPLI for all pairwise connections) for each participant. We assessed whether network strength was correlated with performance on EVT and PPVT, for children born preterm, and for all participants in the study.

### 2.9 MEG effective connectivity analyses

Within the 22–25 Hz frequency range, we evaluated timecourses for information flux (directed—or effective—connectivity) using the phase slope index (PSI) metric (Nolte & Muller, 2010; Nolte et al., 2008). PSI is computed from the complex coherency function for a pair of signals; directionality is determined from phase differences in signals over a specified frequency range. When signal *i* drives signal *j*, the mean phase differences between *i* and *j* will increase with frequency, and PSI will be positive. It is reported as a normalized value, obtained through division by an estimate of standard deviation. This can then be interpreted as any *z*‐statistic. To identify significant connections, data are thresholded at PSI_norm_ ≥ 1.96, which corresponds to the critical value in a two‐tailed normal deviate (*z*) test conducted at alpha = 0.05. Unlike many other metrics of effective connectivity, PSI remains insensitive to mixing/volume conduction, providing robust estimates of information flux between both proximal and distal node pairs.

The primary outcome was a measure of group differences in language representation indexed by differences in localization of blood‐oxygen‐level dependent (BOLD) contrast observed in children born preterm compared to those born at term during task‐based fMRI. Our second outcome measure was comparison of network dynamics, reporting overall patterns of connectivity in children born EPT versus TC on MEG. Finally, we measured the correlation between network topography (fMRI, MEG) and connectivity and observable performance on standardized assessments of language.

## 3 RESULTS

### 3.1 Demographic and behavioral results

EPT (mean gestational age 26 2/7 weeks, range 24 0/7 to 26 6/7 weeks) children had no significant differences in age, sex, or family income (used as a measure of socioeconomic status) compared to TC group. Both groups performed within normal limits on all assessments; however, the TC group scored higher than EPT on the PPVT4, EVT2, and WNV (see Table 1).

**Table 1:** Demographic and neuropsychological data

		Preterm (*n* = 15)	Term (*n* = 15)	*p*
Age (Years ± *SD*)		5.6 ± 0.9	5.7 ± 0.9	*ns*
Sex	Females	6	8	*ns*
	Males	9	7	
Race	White/Caucasian	11	10	*ns*
	Black/African American	2	0	
	Other/Multiple	1	1	
	No response	1	4	
Ethnicity	Hispanic/Latino/a	0	1	*ns*
	Not Hispanic/Latino/a	14	10	
	No response	1	4	
Receptive language	PPVT‐4 (Mean ± *SD*)	116 ± 10	135 ± 9	<.01
Expressive language	EVT‐2 (Mean ± *SD*)	101 ± 10	118 ± 12	<.01
General abilities	WNV (Mean ± *SD*)	105 ± 14	117 ± 10	<.01

Note

Categorical variables were tested using Fisher's Exact Test and *p* values are reported. Continuous variables were tested using *t* tests and *p* values are reported. *SD* = Standard Deviation. PPVT‐4 = Peabody Picture Vocabulary Test. EVT‐2 = Expressive Vocabulary Test. WNV = Wechsler Non‐Verbal Scale of Ability.

### 3.2 fMRI results

Large‐scale activation maps across groups showed the typical pattern of bilateral temporoparietal activation in response to stories versus noise (Figure 1A) reported in prior studies (Holland et al., 2007). Activation on fMRI looked similar between groups. No participant was excluded due to incidental or pathological findings on imaging. No participant was excluded due to excessive motion or inability to complete the task.

### 3.3 MEG results

**Figure 3:**
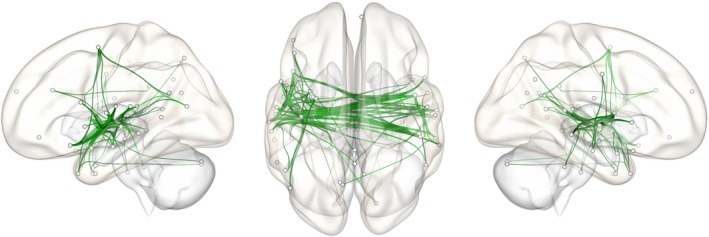
Extremely preterm children show increased functional connectivity in language network. Network “edges” showing significantly increased functional connectivity in EPT versus TC between 22 and 26 Hz during stories listening (observed at various initial thresholds ranging from *t* =1.5 to 3, 10,000 iterations, *p* < .05, corrected for multiple comparisons)

**Figure 4:**
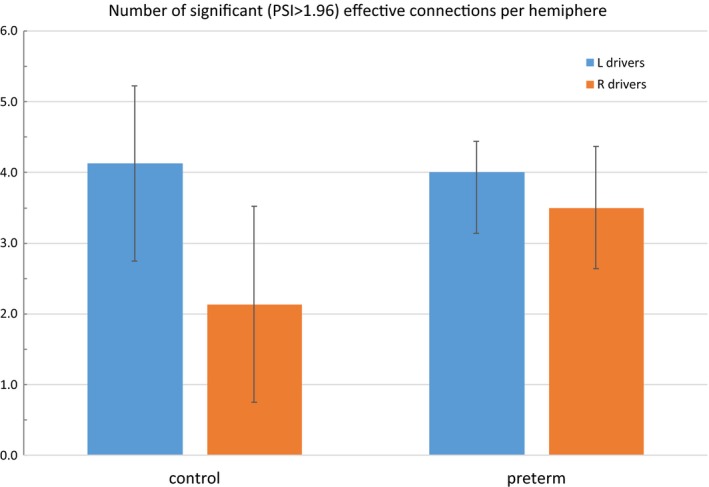
Left and right hemispheric drivers in typically developing controls and children born extremely preterm. Effective connectivity is assessed by the phase slope index (PSI). Data are thresholded at PSInorm ≥ 1.96. The value of ±1.96 corresponds to the critical value in a two‐tailed normal deviate (*z*) test conducted at alpha = 0.05. Surviving significant hemidrivers are subjected to a between‐groups *t* test showing that EPT have significantly more right hemidrivers than TC (*p* < .05)

Analysis of connectivity spectra showed a statistically significant increase in beta‐band wPLI for EPT versus TC. An initial between‐groups *t* test across the spectra revealed a contiguous band of increased functional connectivity from 22 to 25 Hz in EPT (*p* < .05, Figure 2). This was investigated further using Network‐Based Statistics, and EPT had significantly greater network extent (meaning total number of contiguous supra‐threshold connections in the network) than TC at a range of initial statistical thresholds (*t* values) significant at *p* < .05, FWE corrected; the spatial distribution of the significant sub‐network indicates selective involvement of bilateral posterior temporal regions, including Wernicke's area and the right hemisphere homologue (Figure 3). An investigation of the directed, effective connectivity (PSI) revealed more bidirectional information flux in EPT versus TC, with TC having more equal numbers of right and left hemidrivers (sources of directed, effective connectivity; *p* < .05). This suggests that an increase in right hemispheric sources is what is driving the increased bitemporal functional connectivity observed in EPT versus TC (Figure 4). None of the functional or effective connectivity measures on fMRI or MEG were related to scores on neurocognitive measures, suggesting that this is an effect of prematurity and not performance. No participant was excluded due to excessive artifact or inability to complete the task.

## 4 DISCUSSION

Despite improvements in survival of children born extremely preterm, neurocognitive deficits persist in EPT children relative to TC (Younge et al., 2017). These deficits are especially evident in higher‐order processes such as language. This remains a significant public health concern, as EPT and their families require significant healthcare resources while in the NICU and throughout their lives. Our theoretical model was that prematurity would disrupt topography and connectivity of the developing language network and that this would be related to measurable behavior.

Results showed that EPT and TC exhibited the expected pattern of bilateral activation in temporal and parietal areas in response to language stimuli. Conventional analysis of fMRI did not reveal any significant group differences. However, MEG functional and effective connectivity were strikingly different for EPT versus TC. This is significant in that it is not a performance effect or the result of overt brain injury or known neurological deficit. This suggests that it is not the precise “real estate” of regions involved but the connections between these regions which are altered and important for subsequent language development. Thus, investigation of network dynamics—versus traditional analysis of regional alterations in structure and hemodynamics—is a particularly exciting method that might help explain the wide variability of clinical outcomes observed in extremely preterm children and provide us with a biomarker for intervention and prediction.

In the current study, we adopted a fully data‐driven approach for MEG connectivity and network‐based analyses. We intentionally focused on regions shown to support stories listening (fMRI‐constraint) and a narrow frequency band showing significant connectivity differences between groups. Using the fMRI spatial map and MEG connectivity spectra to guide network‐based analyses revealed significant group differences in network topology, in relatively small EPT and TC cohorts. The specific findings reported here do not rule out potentially important language network differences that could occur outside the fMRI‐active region, or at other frequencies. In our previous study of expressive language network connectivity in MEG (Kadis et al., 2016), we observed distinct patterns of connectivity across canonical frequency bands in typically developing controls. Connectivity between the left and right posterior perisylvian region was particularly prominent in alpha, beta, and gamma bands, and absent at lower frequencies. Collectively, findings suggest that the posterior aspects of the pediatric language network, known to support receptive language processes, are dominated by high‐frequency information flux. The significance of this band in the connectivity spectra of language is a novel finding, and it is unclear at this point why it appears to be associated with extreme prematurity in school‐age children. Further study is required.

EPT children with known evidence of brain injury or neurological impairment were excluded and children performed within normal limits on assessments. This phenotype of language network alteration might be positively associated with improved outcomes in a larger preterm population. Future studies are needed—including prospective longitudinal studies and those incorporating a group with known language impairment or structural damage—to further elucidate more beneficial from less beneficial phenotypes, with the aim of developing biomarkers for language network development in EPT. In light of recent evidence that extreme prematurity is not universally devastating for neurological functioning and scholastic performance, investigation into possible markers of resiliency is especially interesting (Garfield et al., 2017). The reported results comparing high‐functioning TC and EPT with no known brain injury are valuable as a step toward that aim. This could help target interventions and refine early prediction of behavioral and school functioning.

The majority of studies looking to combine behavioral, structural, and/or functional modalities feature term‐equivalent neuroimaging and neurocognitive testing at 2 years of age. Our study is unique in inclusion of preterm school‐age children, which is vital to address questions regarding long‐term outcome and academic achievement asked by parents during the NICU stay. No studies known to us combine modalities to elucidate the relationship between brain structure, brain functioning, and neurocognitive performance in preterm children. Our approach is novel in that it seeks to capitalize on the spatial resolution of MRI and temporal resolution of MEG to investigate the topography and connectivity of developing language networks in children who were born preterm. In contrast to these strengths, there are a number of limitations. First, this is a small feasibility study with only 30 children. Second, the language task is a relatively easy, receptive task that was designed so preschool and school‐aged children would be performing at or near ceiling. A more challenging neuroimaging task might reveal more differences in network dynamics. However, we see this as a strength of the study in that we believe it helped ensure that performance was not a confounding variable. Third, applying functional and effective connectivity to language functioning in preterm children is a novel technique for which there is not a standard in the field. However, this is a rapidly growing area of interest, and one that we believe has the potential to capture the neurobiological underpinnings of the insults of prematurity in a way that clinically available structural MRI has failed to do. As novel techniques are applied to help further understand the human connectome of the preterm brain in a collaborative and open‐source manner, new standards will be developed which will enable investigators, physicians, parents, and patients to benefit from knowledge gained.

The reported study combines functional MRI (measure of hemodynamic changes as a proxy for activation) and MEG (more direct measure of fast neuronal activity) methods with standardized neurobehavioral assessments in a developmentally controlled experimental protocol characterizing the neurocognitive status of preterm patients into childhood. We improve on existing studies by having a typical, full‐term control group, excluding children with known injury or deficits, constraining our MEG analyses to regions derived from a validated fMRI language protocol, and using MEG to provide the timecourse of activity within each network node. We consider cognitive function as it relates to various network metrics. These provide a view of the derangements and compensatory mechanisms the brain undergoes to support language function in the context of prematurity, with the ultimate goal of providing biomarkers that can be used to target intervention and health policies to optimize outcomes for our smallest and sickest babies.
